# Type 2 diabetes enhances arterial uptake of choline in atherosclerotic mice: an imaging study with positron emission tomography tracer ^18^F-fluoromethylcholine

**DOI:** 10.1186/s12933-016-0340-6

**Published:** 2016-02-06

**Authors:** Sanna Hellberg, Johanna M. U. Silvola, Max Kiugel, Heidi Liljenbäck, Olli Metsälä, Tapio Viljanen, Jari Metso, Matti Jauhiainen, Pekka Saukko, Pirjo Nuutila, Seppo Ylä-Herttuala, Juhani Knuuti, Anne Roivainen, Antti Saraste

**Affiliations:** Turku PET Centre, University of Turku, Kiinamyllynkatu 4-8, 20520 Turku, Finland; Turku Center for Disease Modeling, University of Turku, Kiinamyllynkatu 10, 20520 Turku, Finland; Genomics and Biomarkers Unit, National Institute for Health and Welfare, Haartmaninkatu 8, 00250 Helsinki, Finland; Department of Pathology and Forensic Medicine, University of Turku, Kiinamyllynkatu 10, 20520 Turku, Finland; Turku PET Centre, Turku University Hospital, Kiinamyllynkatu 4-8, 20520 Turku, Finland; A.I. Virtanen Institute for Molecular Sciences, University of Eastern Finland, Neulaniementie 2, 70210 Kuopio, Finland; Science Service Center, Kuopio University Hospital, Puijonlaaksontie 2, 70210 Kuopio, Finland; Heart Center, Turku University Hospital, Hämeentie 11, 20520 Turku, Finland

**Keywords:** Atherosclerosis, Type 2 diabetes, Positron emission tomography, Inflammation, Choline, Macrophage, ^18^F-fluoromethylcholine, ^18^F-fluorodeoxyglucose

## Abstract

**Background:**

Diabetes is a risk factor for atherosclerosis associated with oxidative stress, inflammation and cell proliferation. The purpose of this study was to evaluate arterial choline uptake and its relationship to atherosclerotic inflammation in diabetic and non-diabetic hypercholesterolemic mice.

**Methods:**

Low-density lipoprotein-receptor deficient mice expressing only apolipoprotein B100, with or without type 2 diabetes caused by pancreatic overexpression of insulin-like growth factor II (IGF-II/LDLR^−/−^ApoB^100/100^ and LDLR^−/−^ApoB^100/100^) were studied. Distribution kinetics of choline analogue ^18^F-fluoromethylcholine (^18^F-FMCH) was assessed in vivo by positron emission tomography (PET) imaging. Then, aortic uptakes of ^18^F-FMCH and glucose analogue ^18^F-fluorodeoxyglucose (^18^F-FDG), were assessed ex vivo by gamma counting and autoradiography of tissue sections. The ^18^F-FMCH uptake in atherosclerotic plaques was further compared with macrophage infiltration and the plasma levels of cytokines and metabolic markers.

**Results:**

The aortas of all hypercholesterolemic mice showed large, macrophage-rich atherosclerotic plaques. The plaque burden and densities of macrophage subtypes were similar in diabetic and non-diabetic animals. The blood clearance of ^18^F-FMCH was rapid. Both the absolute ^18^F-FMCH uptake in the aorta and the aorta-to-blood uptake ratio were higher in diabetic than in non-diabetic mice. In autoradiography, the highest ^18^F-FMCH uptake co-localized with macrophage-rich atherosclerotic plaques. ^18^F-FMCH uptake in plaques correlated with levels of total cholesterol, insulin, C-peptide and leptin. In comparison with ^18^F-FDG, ^18^F-FMCH provided similar or higher plaque-to-background ratios in diabetic mice.

**Conclusions:**

Type 2 diabetes enhances the uptake of choline that reflects inflammation in atherosclerotic plaques in mice. PET tracer ^18^F-FMCH is a potential tool to study vascular inflammation associated with diabetes.

**Electronic supplementary material:**

The online version of this article (doi:10.1186/s12933-016-0340-6) contains supplementary material, which is available to authorized users.

## Background

Diabetes is associated with an increased risk of atherosclerotic cardiovascular disease (CVD), and it is common in patients with known or at risk of CVD [1]. Various factors, including increased oxidative stress and subsequent activation of pro-inflammatory pathways, as well as increased cell proliferation in the arterial wall, contribute to accelerated atherosclerosis in diabetes [2]. Inflammation is a key factor in the pathogenesis of atherosclerosis and its complications [3–5]. Positron emission tomography (PET) with a glucose analogue 2-deoxy-2-[^18^F]-fluoro-*d*-glucose (^18^F-FDG) is a feasible method for non-invasive imaging of atherosclerotic plaque inflammation in large arteries. Vascular uptake of ^18^F-FDG has also been observed in individuals without symptoms of CVD, in the presence of type 2 diabetes mellitus (T2DM) [6, 7], metabolic syndrome [8] and obesity [9]. Accumulation of ^18^F-FDG in atherosclerotic plaques has been attributed to high glucose uptake in macrophages [4, 10]. In addition to the number of macrophages, ^18^F-FDG uptake may reflect their polarization [11, 12] or augmentation of glucose uptake induced by hypoxia [13, 14]. Furthermore, high blood glucose levels in T2DM can diminish ^18^F-FDG uptake in atherosclerosis [7]. These factors may complicate the interpretation of ^18^F-FDG signal from arteries, especially in diabetic individuals, and therefore, alternative tracers for the quantification of inflammation in atherosclerosis have been investigated [15–17].

Choline is taken up into cells by choline transporters, phosphorylated by choline kinase, further metabolized to phosphatidylcholine, and eventually incorporated into the cell membrane or used for lipoprotein assembly. Radiolabeled derivatives of choline have been utilized to detect increased choline uptake in tumor cells [18, 19] and in macrophages at the sites of inflammation [20, 21]. Previous studies have shown increased accumulation of radiolabeled choline derivatives in macrophage-rich atherosclerotic lesions [22, 23] or aortic aneurysms [24]. In asymptomatic patients, ^11^C-choline or ^18^F-fluoromethylcholine (^18^F-FMCH) uptake has been detected in large vessels, predominantly in non-calcified atherosclerotic lesions [25, 26]. We hypothesized that the pro-inflammatory phenotype in T2DM is associated with increased choline uptake, thus making ^18^F-FMCH a potential tracer for monitoring the progression of atherosclerosis in diabetes.

For this study, we evaluated the uptake of ^18^F-FMCH and ^18^F-FDG in inflamed atherosclerotic plaques in non-diabetic and diabetic hypercholesterolemic mice [27]. We studied the kinetics and biodistribution of ^18^F-FMCH by PET/computed tomography (CT) imaging and in ex vivo tissue samples. Uptake of ^18^F-FMCH or ^18^F-FDG in atherosclerotic aorta was first quantified by gamma counting and then compared between plaques and adjacent normal vessel wall by autoradiography of aortic tissue sections. Furthermore, ^18^F-FMCH uptake was compared with the extent and phenotype of macrophages in atherosclerotic plaques, the plasma levels of inflammatory mediators and metabolic markers.

## Methods

### Animals and study design

We utilized two hypercholesterolemic mouse models in the study. LDLR^−/−^ApoB^100/100^ mice are deficient in low-density lipoprotein (LDL) receptor and express only apolipoprotein B100 (strain #003000, The Jackson Laboratory, Bar Harbor, ME, USA). IGF-II/LDLR^−/−^ApoB^100/100^ mice (A. I. Virtanen Institute for Molecular Sciences, University of Eastern Finland, Kuopio, Finland) have a similar lipid profile as LDLR^−/−^ApoB^100/100^, and additionally represent the characteristics of T2DM (insulin resistance and impaired glucose tolerance in the presence of mildly elevated fasting glucose levels) due to the overexpression of insulin-like growth factor II (IGF-II) in pancreatic beta cells [27, 28]. The mice were fed, for a period of 4 months, with a high-fat diet (TD.88137 adjusted calories diet, Harlan Teklad, 42 % of calories from fat, 0.2 % total cholesterol, no sodium cholate, Harlan Laboratories, Madison, WI, USA) to accelerate atherosclerosis development.

Accumulation of ^18^F-FMCH in atherosclerotic plaques was compared in LDLR^−/−^ApoB^100/100^ and IGF-II/LDLR^−/−^ApoB^100/100^ mice (*n* = 11/group) at the age of 6–7 months, after 4 months on high-fat diet. For comparison, age- and gender-matched groups of mice (*n* = 11–12/group) were similarly studied using ^18^F-FDG. Healthy C57BL/6N mice fed with normal chow diet (age 8 months, *n* = 13) were studied with ^18^F-FMCH as controls. The characteristics of the study animals are shown in Table 1. Data on additional 8–10-month-old IGF-II/LDLR^−/−^ApoB^100/100^ mice fed with a longer high-fat diet are shown in the Additional file 1.

**Table 1:** Characteristics of the study animals

Tracer	Mouse strain	N of mice (m/f)	Weight (g)	Age (months)	High-fat diet (months)	Injected radioactivity (MBq)	ARG (*n*)
^18^F-FMCH	LDLR^−/−^ApoB^100/100^	11 (7/4)	33.2 ± 6.0	6.5 ± 0.1	4.4 ± 0.0	9.4 ± 0.9	8
	IGF-II/LDLR^−/−^ApoB^100/100^	11 (6/5)	32.4 ± 6.8	6.4 ± 0.3	3.9 ± 0.5	10.3 ± 0.6	11
	C57BL/6N	13 (9/4)	38.2 ± 7.9	8.0 ± 2.6	NA	11.3 ± 2.2	6
	PET/CT IGFII/LDLR^−/−^ApoB^100/100^	3 (0/3)	25.0 ± 3.1	4.6 ± 0.0	2.8 ± 0.0	5.7 ± 0.5	3
^18^F-FDG	LDLR^−/−^ApoB^100/100^	12 (8/4)	35.8 ± 8.8	6.6 ± 0.3	4.4 ± 0.2	11.2 ± 1.8	12
	IGF-II/LDLR^−/−^ApoB^100/100^	11 (6/5)	34.7 ± 7.6	6.5 ± 0.4	4.2 ± 0.3	10.8 ± 1.4	11

*LDLR* ^*−/−*^ *ApoB* ^*100/100*^ non-diabetic atherosclerotic mice, *IGF-II/LDLR* ^*−/−*^ *ApoB* ^*100/100*^ diabetic atherosclerotic mice; *C57BL/6N* healthy control mice; *PET/CT* positron emission tomography/computed tomography; *ARG* autoradiography; *NA* not applicable. The data are expressed as mean ± SD

All the animal experiments were performed in accordance with the relevant European Union Directive and approved by the National Animal Experiment Board in Finland and the Regional State Administrative Agency for Southern Finland (Licence numbers ESAVI/1583/04.10.03/2012 and ESAVI/2163/04.10.07/2015). The mice were housed in standardized conditions with a 12/12 h dark/light cycle and they had access to water and food ad libitum. The studies were performed under isoflurane anesthesia. The mice were fasted for 4 h before the ^18^F-FDG injection to standardize plasma glucose level. Since fasting had not shown any detectable effect on choline uptake in previous studies [29] or in our pre-study (see Additional file 1), the mice were not fasted before the ^18^F-FMCH injection.

### Radiotracers

The ^18^F-FMCH batches were purchased from MAP Medical Technologies Oy (Helsinki, Finland). The radiochemical purity exceeded 95 % in every batch. ^18^F-FDG was synthesized in the radiopharmaceutical laboratory of Turku PET Centre. ^18^F-FMCH in vivo stability was assessed by radio-HPLC (see Additional file 1).

### In vivo PET/CT imaging

In order to study the distribution kinetics of ^18^F-FMCH, three IGF-II/LDLR^−/−^ApoB^100/100^ mice were imaged with Inveon Multimodality small animal PET/CT scanner (Siemens Medical Solutions, Knoxville, TN, USA). The mice were injected with approximately 6 MBq of ^18^F-FMCH via tail vein and a dynamic 30-minute PET was started at the same time. After PET imaging, 100–150 µl of intravenous contrast agent (eXia 160XL, Binitio Biomedical, Ottawa, Ontario, Canada) was injected and a high-resolution CT imaging was performed. The CT acquisition consisted of 270 projections with the exposure time of 1250 ms for a full 360° rotation. X-ray voltage was 80 kVp and anode current 500 µA.

PET data acquired in a list mode were iteratively reconstructed with a 2-dimensional ordered-subsets expectation maximization algorithm into 30 × 10 s and 15 × 60 s frames. A reconstructed image had 128 × 128 × 159 matrix size with a pixel size of 0.776 × 0.776 × 0.796 mm. CT images were reconstructed with a filtered back-projection algorithm (pixel size 0.094 × 0.094 × 0.094 mm). In vivo PET and CT images were co-registered and dynamic PET data was analyzed with Carimas 2.8 software (Turku PET Centre, Turku, Finland). Regions of interest (ROI) were defined in selected tissues based on the high-resolution CT in order to obtain time-activity curves. The ROI for blood pool was located in vena cava.

### Ex vivo PET imaging

For a subset of mice studied with ^18^F-FMCH (3 LDLR^−/−^ApoB^100/100^ and 3 C57BL/6N mice), an ex vivo PET was performed for the aorta and heart. The mice were killed at 20 min post-injection, the aortas and hearts were excised, measured for radioactivity and positioned into a tube filled with ultrasonography gel. The tube was placed in the PET camera (Inveon Multimodality small animal PET/CT scanner, Siemens Medical Solutions, Knoxville, TN, USA) and static 40-minute min PET was performed. PET data were reconstructed with a 3-dimensional ordered-subsets expectation maximization algorithm. A reconstructed image had 128 × 128 × 159 matrix size with a pixel size of 0.776 × 0.776 × 0.796 mm.

### Blood sampling and ex vivo tracer distribution measurement

The mice were i.v. injected with 10–11 MBq of ^18^F-FMCH or ^18^F-FDG and killed with cardiac puncture and cervical dislocation under deep anesthesia at 20 or 90 min post-injection, respectively. The time points were chosen based on tracer-specific kinetics [23, 30]. Approximately 50 µl of blood was measured for radioactivity. The rest of the blood sample was centrifuged and the separated plasma was stored at −70 °C for further analyses. To assess the distribution of the radiotracers, the aortas and selected other tissues were excised, weighed and measured using a gamma counter (1480 Wizard 3″; Perkin Elmer/Wallac, Turku, Finland or Triathler 3″, Hidex, Turku, Finland). Radioactivity concentration was decay-corrected to the injection time and expressed as percentage of injected radioactivity dose per gram of tissue (% IA/g).

### Autoradiography of aortic sections

The distribution of ^18^F-FMCH and ^18^F-FDG in the aortas of mice was further studied by autoradiography. The aortas were frozen in ice-cold isopentane, cut into sequential 20- and 8-µm sections and apposed to an imaging plate for digital autoradiography. After an overnight exposure, the plates were scanned with Fuji Analyzer BAS-5000 (Fuji, Tokyo, Japan; internal resolution 25 μm) and the sections were stored at −70 °C.

The autoradiography analysis was performed with TINA 2.1 software (Raytest Isotopemessgeräte, GmbH, Straubenhardt, Germany). In the autoradiographs of the 20-µm sections, ROIs were defined in plaques (excluding calcifications), adjacent histologically normal vessel wall and vessel-surrounding adventitia, based on hematoxylin-eosin staining (H&E). Background counts were subtracted and results expressed as photo-stimulated luminescence (PSL)/mm^2^. The values were normalized for injected radioactivity, mouse weight and radioactivity decay during exposure. On average, 50 ROIs for plaque, 25 for wall and 27 for adventitia per animal were analyzed. For atherosclerotic mice, plaque-to-wall uptake ratio was calculated.

### Histology and immunohistochemistry

The 20-µm aortic sections were stained with H&E for histological evaluation. In order to assess the plaque inflammation, the 8-µm sections were immunostained for macrophages with anti-Mac-3 antibody (clone M3/84, 1:5000, BD Pharmingen, Franklin Lakes, NJ, USA) as described before [31]. Additional 8-µm sections were stained with anti-Ki-67 antibody (clone TEC-3, M7249, 1:1000, Dako, Glostrup, Denmark) to detect cell proliferation. Aortic roots (*n* = 8–9/group) were formalin-fixed, paraffin-embedded, and cut into serial 5-µm sections for the histological comparison of the characteristics of atherosclerosis in LDLR^−/−^ApoB^100/100^ and IGF-II/LDLR^−/−^ApoB^100/100^ mice. The sections were stained with Movat’s pentachrome to assess the plaque burden, expressed as intima-to-media ratio (IMR). Macrophages were stained with anti-Mac-3 antibody. Additionally, sections were stained with anti-inducible nitric oxide synthase (iNOS) or anti-mannose receptor C-type 1 (MRC-1) antibodies to detect M1 (pro-inflammatory) and M2 (anti-inflammatory) polarized macrophages, respectively. Immunohistochemical staining for scavenger receptor CD36, involved in the atherogenesis, was also performed. The staining protocols are described in Additional file 1 and the analyses in [32].

### Comparison of ^18^F-FMCH uptake and macrophages in plaques

To compare ^18^F-FMCH uptake and inflammation (amount of macrophages), a subset of diabetic mice (*n* = 8) was studied. In autoradiographs, ROIs were defined in plaque areas showing no or only few macrophages and areas with macrophage infiltration. Based on the areal percentage of Mac-3-positive staining as quantified with ImageJ software (Fiji, National Institutes of Health, Bethesda, MD, USA), the plaque areas were classified as representing low, intermediate or high macrophage density. ^18^F-FMCH uptake in the areas was expressed as PSL/mm^2^ as described above.

### Correlation of ^18^F-FMCH plaque uptake and plasma biomarkers

Plasma samples from eleven LDLR^−/−^ApoB^100/100^, eleven IGF-II/LDLR^−/−^ApoB^100/100^ and nine C57BL/6N mice were analyzed for lipids and lipid-associated proteins (total cholesterol, phospholipids, triglycerides, phospholipid transfer protein [PLTP] and paraoxonase-1 [PON-1]), metabolic markers (glucagon, glucose, C-peptide, insulin and leptin), and inflammatory mediators (interferon-γ [IFN-γ], interleukin-6 [IL-6], monocyte chemoattractant protein-1 [MCP-1] and cytokine regulated on activation, normal T cell expressed and secreted [RANTES]) (see Additional file 1). The markers in plasma were plotted against the mean ^18^F-FMCH uptake in plaques measured by autoradiography (PSL/mm^2^) in each mouse.

### Statistical analyses

Results are expressed as mean ± SEM unless otherwise specified. Statistical analyses were conducted with IBM SPSS Statistics 21 (IBM Corp., Armonk, NY, USA). The comparisons between two groups were made using independent samples *t* test, and the comparisons between multiple groups using one-way ANOVA with Tukey or Tamhane correction. Paired t-test was applied when comparing uptake between different tissues in the same animals. The correlations were assessed with Pearson’s coefficient (r). The *p* values <0.05 were considered statistically significant.

## Results

Total cholesterol levels were similar in non-diabetic (LDLR^−/−^ApoB^100/100^) and diabetic (IGF-II/LDLR^−/−^ApoB^100/100^) mice (31 ± 4.3 and 36 ± 12 mmol/l, respectively), as were also the fasting glucose levels (7.8 ± 1.0 and 9.1 ± 3.1 mmol/l, respectively). There was a tendency towards higher blood levels of insulin, C-peptide and IL-6 in diabetic mice (Additional file 1: Table S3). The total cholesterol was significantly lower in C57BL/6N control than in the hypercholesterolemic mice (1.8 ± 0.3, *p* < 0.001).

**Fig. 1:**
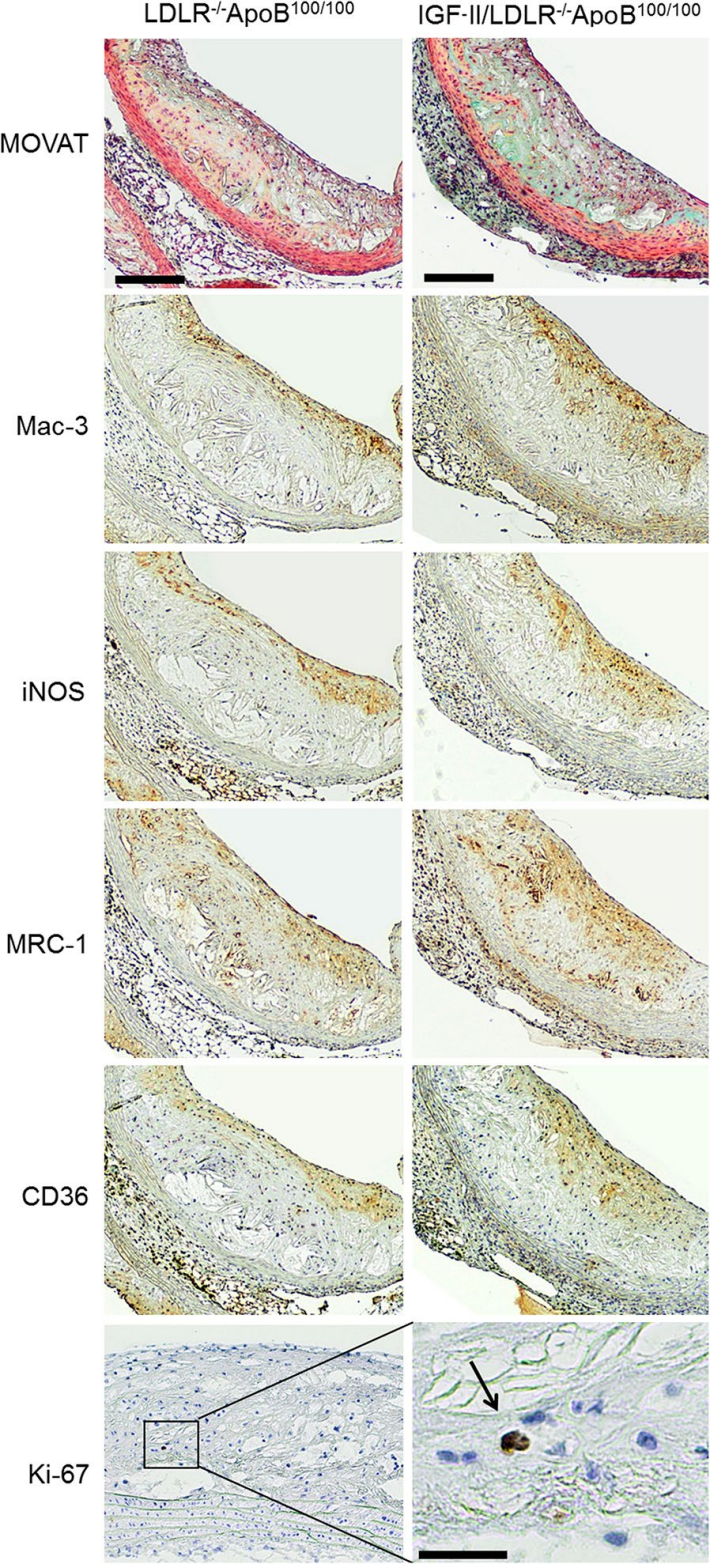
Histology of atherosclerotic plaques in non-diabetic LDLR^−/−^ApoB^100/100^ and diabetic IGF-II/LDLR^−/−^ApoB^100/100^ mice. The sections are from aortic roots (*scale bar* 100 µm) except for Ki-67, which is from aortic arch (*scale bar* for inset 20 µm). Movat: histology (*black* = nuclei; *yellow* = collagen, reticular fibers; *blue* = ground substance, mucin; *red* = muscle), Mac-3: macrophages, iNOS: M1 polarized macrophages, MRC-1: M2 polarized macrophages, CD36: macrophage scavenger receptor, Ki-67: proliferative cells

The aortas of both non-diabetic and diabetic hypercholesterolemic mice showed extensive atherosclerosis, mainly fibroatheroma-type lesions with large, confluent areas of Mac-3-positive macrophages, whereas no atherosclerosis was observed in control mice (Fig. 1). All plaques showed positivity for iNOS and MRC-1, indicating M1 and M2 polarized macrophages, respectively. The scavenger receptor CD36 co-localized with the macrophage-positive areas of plaques, while only occasional Ki-67 positive cells were observed. Occasional plaque calcifications were seen in 60 % of non-diabetic and 73 % of diabetic mice. The plaques of non-diabetic and diabetic mice showed no difference in any of the measured histological characteristics (Table 2).

**Table 2:** Quantitative results of histology in non-diabetic (LDLR^−/−^ApoB^100/100^) and diabetic (IGF-II/LDLR^−/−^ApoB^100/100^) atherosclerotic mice

	LDLR^−/−^ApoB^100/100^ (*n* = 8)	IGF-II/LDLR^−/−^ApoB^100/100^ (*n* = 9)
Intima-to-media ratio (plaque size)	2.7 ± 0.88	1.8 ± 0.51
Mac-3 positive area (%)	15 ± 2.3	17 ± 0.98
iNOS positive area (%)	14 ± 1.3	14 ± 1.7
MRC-1 positive area (%)	30 ± 1.3	32 ± 1.2
CD36 positive area (%)	24 ± 1.6	28 ± 0.66

In addition to plaque size (intima-to-media ratio), areal percentages of macrophages (Mac-3), M1 polarized macrophages (iNOS), M2 polarized macrophages (MRC-1) and scavenger receptor (CD36) in atherosclerotic lesions in the aortic root are shown. The data are expressed as mean ± SEM. No statistically significant differences were observed between the mouse strains

### In vivo PET/CT imaging

**Fig. 2:**
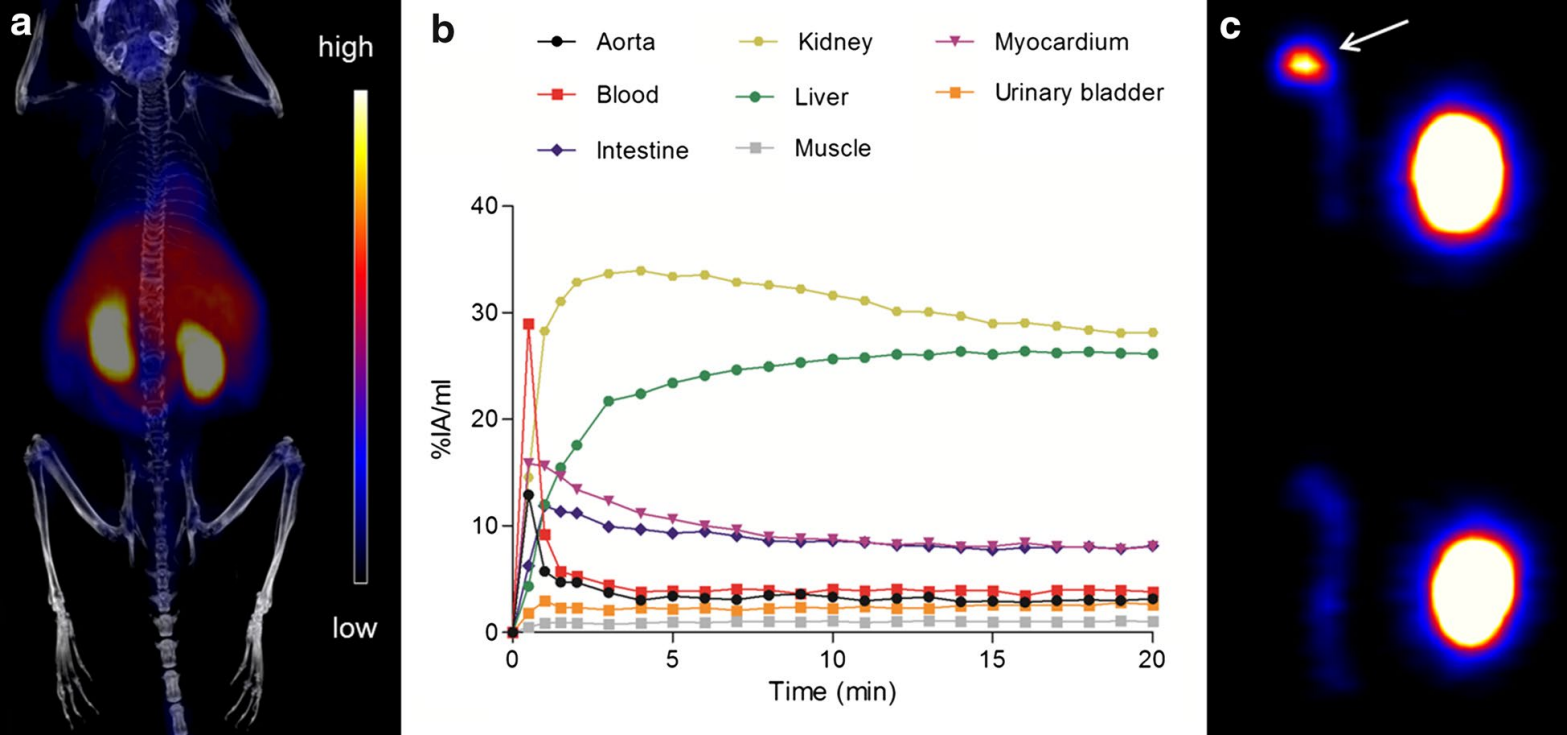
In vivo and ex vivo PET Imaging. **a** Whole-body ^18^F-FMCH PET/CT image of an IGF-II/LDLR^−/−^ApoB^100/100^ mouse. **b** Mean time-activity curves of radioactivity in selected tissues from three mice. **c** Representative ^18^F-FMCH PET image of the aorta (*left*) and heart (*right*) of atherosclerotic LDLR^−/−^ApoB^100/100^ and healthy C57BL/6N mice. High focal uptake can be seen in the aortic arch of the LDLR^−/−^ApoB^100/100^ mouse (*arrow*)

Figure 2a, b shows a representative whole-body ^18^F-FMCH PET/CT image and average time-activity curves of selected tissues in three atherosclerotic mice. ^18^F-FMCH radioactivity was cleared from the blood rapidly after injection and the uptake in tissues reached a plateau between 15 and 20 min post-injection. Therefore, a 20 min time point was chosen for ex vivo studies. The highest radioactivity concentrations were observed in kidneys and liver.

Ex vivo ^18^F-FMCH PET imaging revealed focal areas of high tracer uptake in the aortic arch of atherosclerotic mice, as compared with the lower and homogeneous uptake in the aortas of healthy control mice. PET images of the aorta and the heart are shown in Fig. 2c.

### Uptake of ^18^F-FMCH in diabetic atherosclerosis

The uptakes of ^18^F-FMCH in aorta and other tissues are shown in Table 3. Aortic ^18^F-FMCH uptake was higher in diabetic than non-diabetic atherosclerotic mice or healthy controls (*p* = 0.025 and *p* < 0.001, respectively). The remaining blood radioactivity was lower in diabetic than non-diabetic mice (*p* = 0.0050), and thus, the aorta-to-blood ratio was higher (*p* = 0.017). The radioactivity concentrations in urine also tended to be lower in diabetic than non-diabetic mice (*p* = 0.058).

**Table 3:** Ex vivo distribution of ^18^F-FMCH

	LDLR^−/−^ApoB^100/100^	IGF-II/LDLR^−/−^ApoB^100/100^	C57BL/6N
Aorta	1.5 ± 0.11	2.0 ± 0.17^a, b^	1.2 ± 0.094
Blood	0.98 ± 0.076	0.58 ± 0.066^b^	0.84 ± 0.090
Intestine	8.1 ± 0.75	11 ± 2.0	8.7 ± 2.9
Kidney	49 ± 2.8	41 ± 5.1	37 ± 3.2
Liver	19 ± 2.8	13 ± 2.0	18 ± 2.9
Lungs	11 ± 0.48	13 ± 1.0^a^	9.2 ± 0.77
Muscle	0.91 ± 0.086	1.2 ± 0.10	0.86 ± 0.12
Myocardium	6.8 ± 0.44	8.4 ± 0.80	6.3 ± 0.64
Pancreas	7.7 ± 0.78	8.1 ± 0.86	6.0 ± 0.42
Spleen	6.6 ± 0.30	6.7 ± 0.77	5.0 ± 0.61
Urine	20 ± 5.4	6.3 ± 2.1	15 ± 4.0
White adipose tissue	0.50 ± 0.12	0.51 ± 0.12	0.29 ± 0.040

The distribution of ^18^F-FMCH is measured at 20 min post-injection in non-diabetic (LDLR^−/−^ApoB^100/100^) and diabetic (IGF-II/LDLR^−/−^ApoB^100/100^) atherosclerotic mice and in healthy (C57BL/6N) controls. The results are expressed as percentage of injected radioactivity dose per gram of tissue (mean ± SEM)

^a^a significant difference as compared with C57BL/6N mice

^b^a significant difference as compared with LDLR^−/−^ApoB^100/100^ mice

**Fig. 3:**
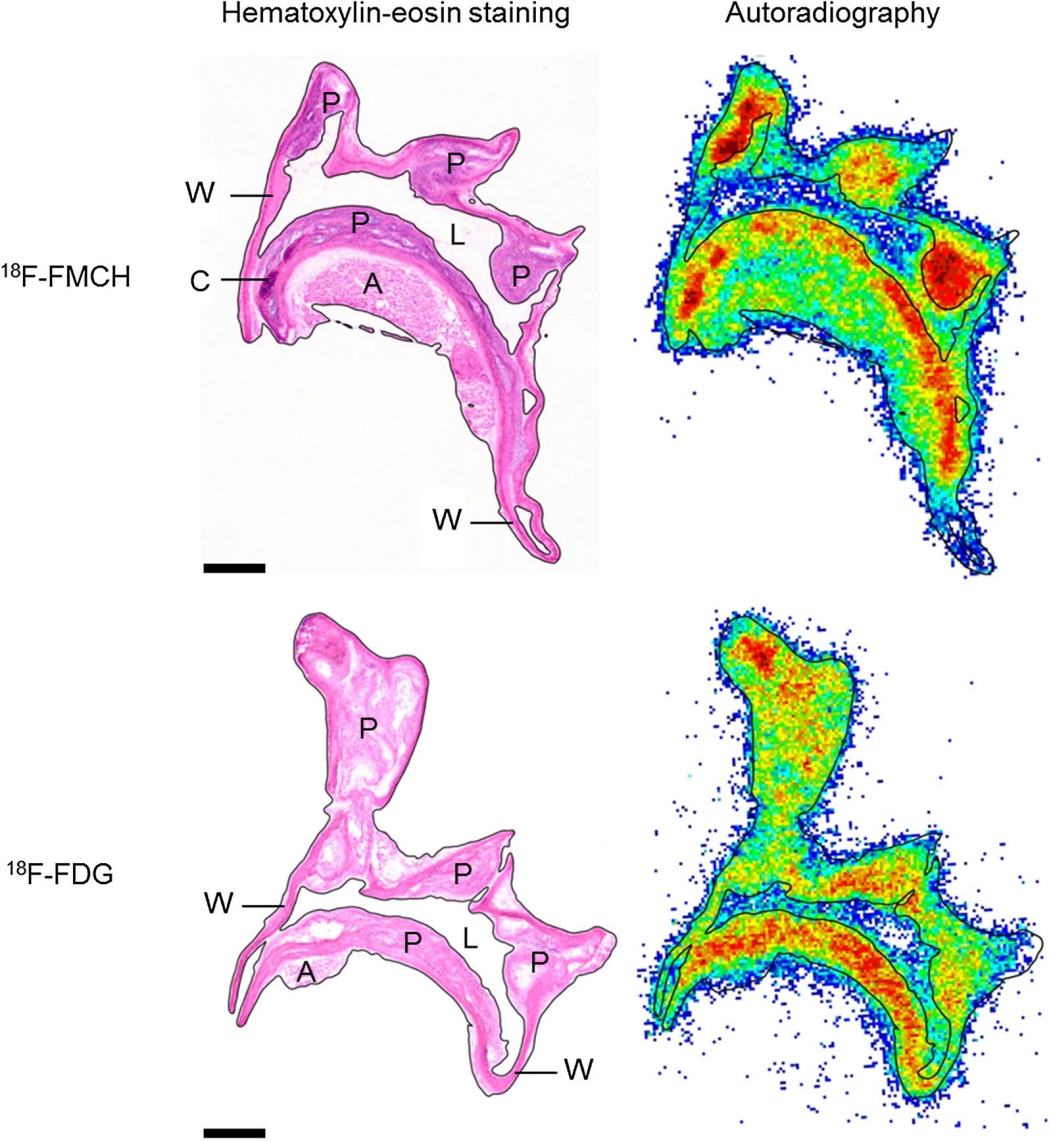
Histology and ^18^F-FMCH and ^18^F-FDG autoradiography of atherosclerotic aortas in diabetic IGF-II/LDLR^−/−^ApoB^100/100^ mice. *Scale bar* 500 µm. Both tracers show focal uptake in the atherosclerotic plaques (P) in the aortic arch and its branches. *W* normal vessel wall, *A* adventitia, *C* calcification, *L* lumen

Autoradiography showed strong and clearly delineated accumulation of ^18^F-FMCH in atherosclerotic plaques in both diabetic and non-diabetic mice (Fig. 3; Table 4). ^18^F-FMCH uptake was significantly higher in plaques than in normal vessel wall or adventitia. In the normal vessel wall, uptake of ^18^F-FMCH was similar in non-diabetic and diabetic atherosclerotic mice and in controls. ^18^F-FMCH uptake in plaques was higher in diabetic mice, but the plaque-to-wall ratios were similar in non-diabetic and diabetic mice (2.5 ± 0.10 and 2.6 ± 0.085, *p* = 0.19).

**Table 4:** Aortic uptake of ^18^F-FMCH and ^18^F-FDG in ex vivo autoradiography

	^18^F-FMCH			^18^F-FDG	
	LDLR^−/−^ApoB^100/100^	IGF-II/LDLR^−/−^ApoB^100/100^	C57BL/6N	LDLR^−/−^ApoB^100/100^	IGF-II/LDLR^−/−^ApoB^100/100^
Adventitia	140 ± 9.1	160 ± 12	100 ± 8.4^a^	210 ± 39	130 ± 12
Wall	76 ± 5.8	89 ± 6.8	69 ± 7.5	160 ± 18	140 ± 12
Plaque	190 ± 11	240 ± 20^a^	NA	320 ± 30	290 ± 20
Plaque-to-wall ratio	2.5 ± 0.10	2.6 ± 0.085	NA	2.1 ±0.16	2.1 ± 0.16
Plaque vs. wall *p* value	<0.001	<0.001	NA	0.0023	<0.001
Plaque vs. adventitia *p* value	0.018	0.022	NA	0.061	<0.001

Uptake of the tracers in different parts of vascular tissue are measured in non-diabetic (LDLR^−/−^ApoB^100/100^) and diabetic (IGF-II/LDLR^−/−^ApoB^100/100^) atherosclerotic mice and in healthy (C57BL/6N) controls. The results are expressed as count densities (photo-stimulated luminescence per mm^2^, mean ± SEM).

*NA* not applicable

^a^a significant difference as compared with LDLR^−/−^ApoB^100/100^ mice

### Comparison of ^18^F-FMCH plaque uptake and macrophages in immunohistochemistry

**Fig. 4:**
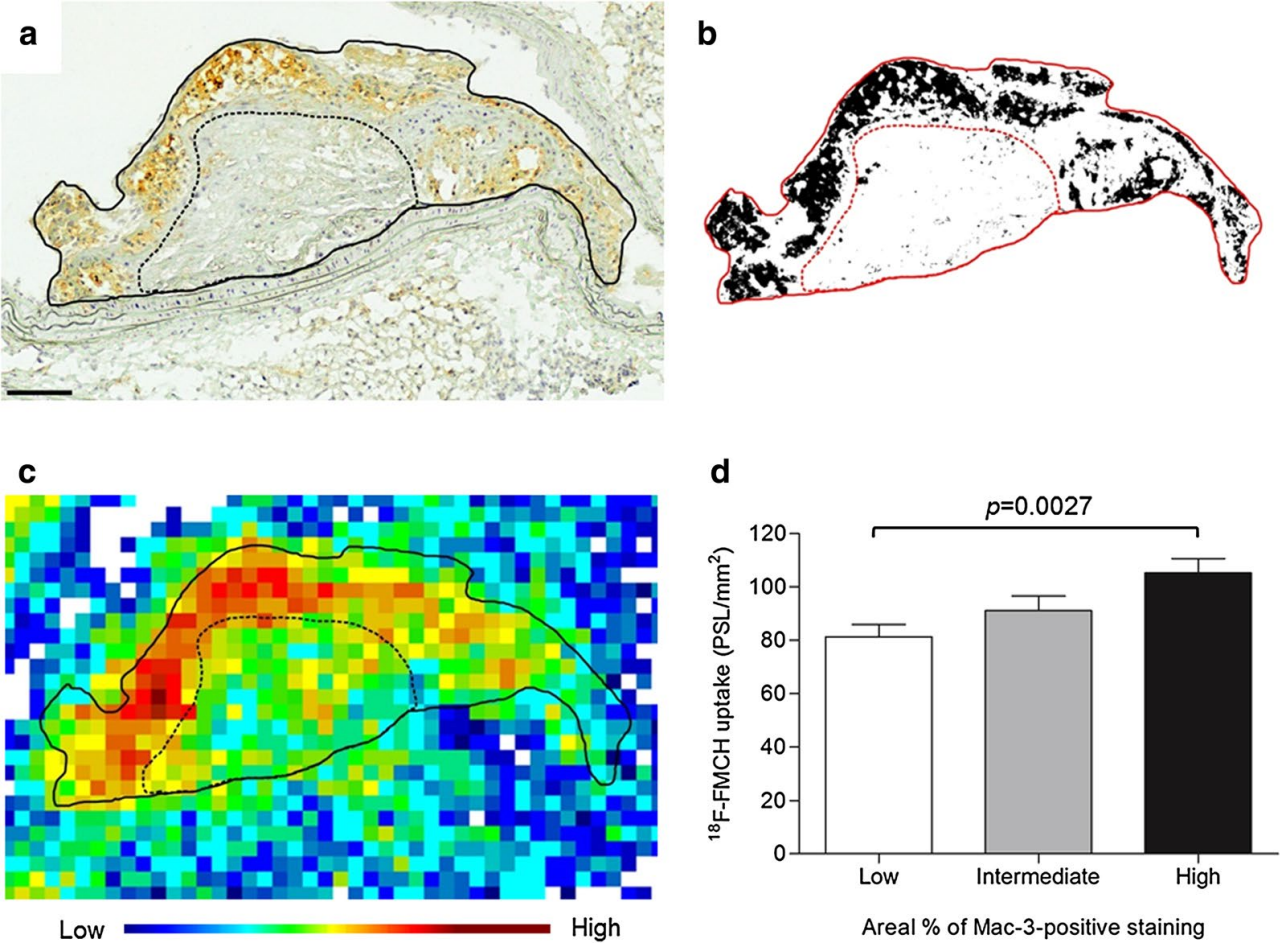
^18^F-FMCH uptake and the density of macrophages in atherosclerotic plaques in diabetic IGF-II/LDLR^−/−^ApoB^100/100^ mice. **a** Macrophage immunostaining (Mac-3, brown) of aortic plaque. The dotted line separates areas of visually high and low macrophage density. *Scale bar* 100 µm. **b** Binary image from ImageJ software shows Mac-3-positive staining as black, and the areal percentage of positive staining is measured. **c** Autoradiograph of the plaque shows high radioactivity co-localizing with the Mac-3-positive staining. **d** Quantitative uptake of ^18^F-FMCH in plaque areas with low, intermediate or high areal percentage of Mac-3-positive staining

The uptakes of ^18^F-FMCH were compared in plaque areas with low, intermediate or high areal percentage of Mac-3 staining. The obtained average areal percentages were 12, 38 and 52 %, respectively. The uptake of ^18^F-FMCH was significantly higher in areas with high than low areal percentage of Mac-3-staining (110 ± 5.3 vs. 81 ± 4.5 PSL/mm^2^, *p* = 0.0027, Fig. 4).

### Correlation of ^18^F-FMCH plaque uptake and plasma biomarkers

**Fig. 5:**
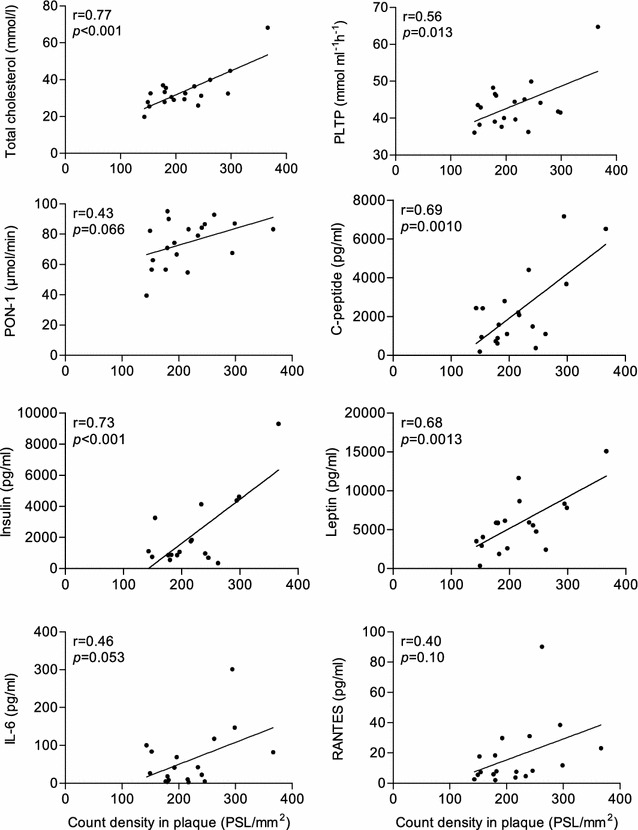
Correlation of ^18^F-FMCH uptake and plasma levels of lipids, metabolic markers and cytokines. Pooled data from non-diabetic LDLR^−/−^ApoB^100/100^ and diabetic IGF-II/LDLR^−/−^ApoB^100/100^ mice. *PLTP* phospholipid transfer protein; *PON-1* paraoxonase-1; *IL-6* interleukin-6*; RANTES* cytokine regulated on activation, normal T cell expressed and secreted

The average ^18^F-FMCH uptake in plaques (pooled data from non-diabetic and diabetic mice) showed positive correlation with plasma levels of total cholesterol (r = 0.77, *p* < 0.001), PLTP (r = 0.56, *p* = 0.013), C-peptide (r = 0.69, *p* = 0.0010), insulin (r = 0.73, *p* < 0.001) and leptin (r = 0.68, *p* = 0.0013). A trend towards positive correlation was observed with plasma PON-1 activity (r = 0.43, *p* = 0.066), whereas no correlation was observed with plasma glucose (r = −0.25, *p* = 0.32). There was a trend towards positive correlation between the uptake of ^18^F-FMCH in plaques and the blood levels of IL-6 (r = 0.46, *p* = 0.053) and RANTES (r = 0.40, *p* = 0.10). No correlation was observed to other measured markers. The correlation plots are shown in Fig. 5 and Supplemental Fig. 4 in Additional file 1.

### Comparison of ^18^F-FMCH and ^18^F-FDG uptakes in the atherosclerotic aorta

**Fig. 6:**
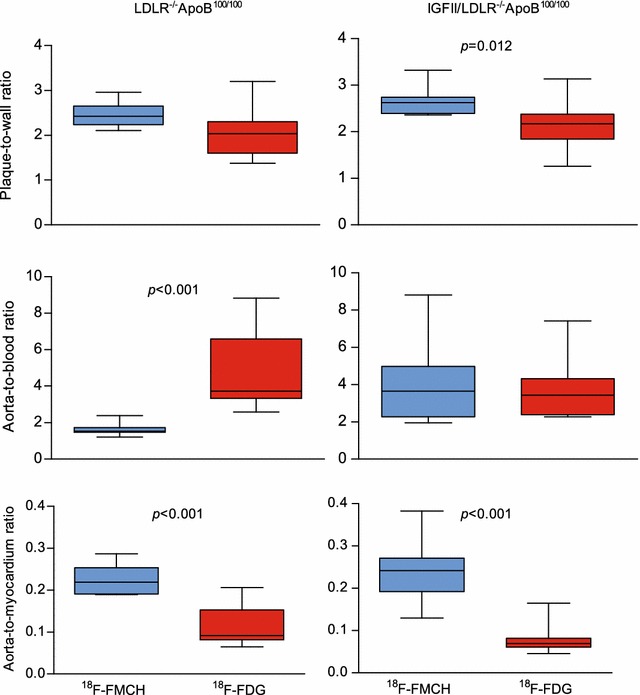
Comparison of ^18^F-FMCH and ^18^F-FDG target-to-background ratios in non-diabetic LDLR^−/−^ApoB^100/100^ and diabetic IGF-II/LDLR^−/−^ApoB^100/100^ atherosclerotic mice. The plaque-to-wall uptake ratio measured from autoradiography is higher with ^18^F-FMCH as compared with ^18^F-FDG in diabetic mice. The ^18^F-FMCH aorta-to-blood ratio measured ex vivo by gamma counting is lower as compared with ^18^F-FDG in non-diabetic mice. ^18^F-FMCH shows a significantly higher aorta-to-myocardium ratio in both mouse strains

Representative ^18^F-FDG and ^18^F-FMCH autoradiographs of the mouse aortic arch are shown in Fig. 3. Average plaque-to-wall ratios were similar between ^18^F-FMCH and ^18^F-FDG in non-diabetic mice, but significantly higher with ^18^F-FMCH than ^18^F-FDG in diabetic mice (Fig. 6). Gamma counting showed that the absolute uptake of ^18^F-FMCH in the whole aorta was lower than that of ^18^F-FDG in both diabetic (2.0 ± 0.17 vs. 4.7 ± 0.41 % IA/g, *p* < 0.001) and non-diabetic mice (1.5 ± 0.11 vs. 5.0 ± 0.40, *p* < 0.001). However, the aorta-to-blood ratios of the tracers were similar in diabetic mice (3.9 ± 0.65 vs. 3.6 ± 0.49, *p* = 0.70), whereas in non-diabetic mice, the ratio was lower with ^18^F-FMCH than with ^18^F-FDG (1.6 ± 0.14 vs. 4.9 ± 0.68, *p* < 0.001). The uptake of ^18^F-FMCH in myocardium was, however, lower than that of ^18^F-FDG in both diabetic (8.4 ± 0.80 vs. 65 ± 8.0 % IA/g, *p* < 0.001) and non-diabetic (6.8 ± 0.44 vs. 49 ± 4.7, *p* < 0.001) mice, resulting in a higher aorta-to-myocardium ratio (Fig. 6).

## Discussion

The uptake of the PET tracer ^18^F-FMCH is increased in the atherosclerotic aorta of mice with T2DM. The uptake in atherosclerotic plaques was associated with the amount of Mac-3 positive macrophages in plaques, as well as the plasma levels of total cholesterol, certain metabolic markers and cytokines. In comparison with ^18^F-FDG, ^18^F-FMCH provided similar or higher target-to-background ratios between atherosclerotic plaques and the normal vessel wall, as well as aorta and blood or myocardium in diabetic mice. These results indicate that T2DM is associated with increased uptake of choline in atherosclerosis and that ^18^F-FMCH may be useful for the evaluation of vascular inflammation in diabetes.

Studies using ^18^F-FDG PET have revealed subclinical vascular inflammation in patients with abnormal glucose metabolism [6–8]. More recent studies have indicated contribution of pericardial and visceral fat to vascular inflammation [9, 33]. Molecular imaging of vascular inflammation is a potential tool to study mechanisms of atherosclerosis, effects of therapies and eventually risk of CVD in high-risk individuals, such as those with diabetes [4].

The LDLR^−/−^ApoB^100/100^ mouse model is a well characterized and widely utilized model of hypercholesterolemia and atherosclerosis. Abnormalities in apolipoprotein E (ApoE) rarely contribute to human hypercholesterolemia, and therefore, the LDLR^−/−^ mouse model better mimics the human disease. The addition of the gene modification leading to the expression of only apolipoprotein B100 on the LDLR^−/−^ background further raises the cholesterol level and amplifies the progression of atherosclerosis [28]. Despite the only mildly elevated fasting glucose levels, the IGF-II/LDLR^−/−^ApoB^100/100^ mice overexpressing IGF-II in pancreatic beta cells represent the insulin resistance and impaired glucose tolerance that is consistent with the T2DM phenotype. Although their lipid profile is similar to that of the LDLR^−/−^ApoB^100/100^, the diabetic mice represent accelerated development of macrovascular complications, including more calcified, less organized and more complex lesions with higher IL-6 expression [27, 34]. Our findings of elevated plasma insulin, C-peptide and IL-6 levels are in line with the T2DM phenotype. Taken together, these data suggest a more pro-inflammatory milieu in the diabetic IGF-II/LDLR^−/−^ApoB^100/100^ mice as compared with the non-diabetic LDLR^−/−^ApoB^100/100^ mice.

The ^18^F-FMCH uptake was increased in the atherosclerotic aorta of diabetic mice as compared with non-diabetic mice. In addition, the correlations between the ^18^F-FMCH plaque uptake and the plasma C-peptide, insulin and leptin levels support the association of the uptake to diabetic phenotype. In both diabetic and non-diabetic mice, the plaques represented similar amounts of M1 and M2 polarized macrophages and also scavenger receptor CD36. Therefore, the higher ^18^F-FMCH uptake in the inflamed plaques may reflect increased choline transport [23] and metabolism in the macrophages. Interestingly, ^18^F-FMCH seems to have an enhanced cellular intake in diabetic mice, since the tracer tends to be more cleared from the blood and less excreted in the urine at the 20-minute time point as compared with non-diabetic mice. The absolute uptake of ^18^F-FMCH was higher in the aortas of diabetic mice as compared with non-diabetic mice, as was also the aorta-to-blood uptake ratio due to lower remaining tracer in circulation. Because blood will always be included in the ROI due to the limited resolution of PET images, high aorta-to-blood ratio is a good property for the in vivo imaging of tracer uptake in atherosclerotic plaques.

In the current study, the plaque-to-wall uptake ratio of ^18^F-FMCH (on average 2.6) was within the same range as that of ^11^C-choline (2.3) or ^18^F-fluorocholine (3.5) in previous studies in mice [22, 23]. In line with previous observations, we discovered that ^18^F-FMCH accumulated in the most inflamed plaques, as shown by high macrophage density. The plaque uptake of ^18^F-FMCH also showed correlation with different metabolic and inflammatory markers. Elevated levels of total cholesterol, PLTP, RANTES, IL-6, insulin and leptin, which showed correlation with ^18^F-FMCH uptake, have been linked with increased atherosclerosis [35–42] thus lending support to the association between ^18^F-FMCH uptake and the risk of atherosclerosis. Recent reports, however, suggest that PLTP activity is inversely correlated with carotid artery disease and linked with PON-1 [43], and that PON-1 is regarded as an atheroprotective enzyme [44, 45].

In previous studies, arterial uptake of ^18^F-FDG or ^11^C-choline did not typically co-localize with calcifications seen on CT supporting the concept that inflammation and calcification occur at different stages of atherosclerosis [4, 25]. Our findings with ^18^F-FMCH suggest the same pattern since vascular tracer uptake was lower in 9-month-old mice (% IA/g 1.7, plaque-to-wall ratio 2.2, see Additional file 1) than in 6-month-old mice (% IA/g 2.0, plaque-to-wall ratio 2.6), despite more extensive vascular calcification (calcifications in all animals at the age of 9 months vs. in 73 % of animals at the age of 6 months), but less macrophages.

Although ^18^F-FDG is the most commonly used tracer for atherosclerotic plaque imaging, the high physiological tracer uptake in the myocardium has limited its application for imaging coronary artery inflammation, although it is possible to reduce the uptake by means of metabolic interventions [4]. In this study, the myocardial uptake was lower for ^18^F-FMCH than for ^18^F-FDG, and, moreover, the myocardial uptake of ^11^C-choline has been negligible in patients [25], thus promoting the feasibility of ^18^F-FMCH in coronary artery imaging. As compared with ^18^F-FDG, ^18^F-FMCH showed superior plaque-to-wall ratio, equal aorta-to-blood ratio and higher aorta-to-myocardium ratio in the diabetic mice, suggesting potential for monitoring atherosclerosis progression in diabetes.

There are some limitations in our study. The reasons for insulin resistance in our mouse model are not fully understood, the model does not represent the full phenotypic spectrum of T2DM in patients, and our findings need to be verified in clinical studies [46]. The feasibility of ^18^F-FMCH for detecting atherosclerotic plaque inflammation by in vivo PET imaging remains to be investigated because, in our study, only young IGF-II/LDLR^−/−^ApoB^100/100^ mice were imaged with PET/CT and it was not possible to visualize the small plaques existing at the young age. Taken together, our findings support the concept of utilizing choline-based radiopharmaceuticals in the imaging of inflammation in diabetic atherosclerosis, but further in vivo imaging studies in patients are needed to proof this.

## Conclusions

The uptake of the radiolabeled choline derivative ^18^F-FMCH was increased by type 2 diabetes in the aortas of atherosclerotic mice and reflected inflammation in atherosclerotic plaques. Comparison with ^18^F-FDG indicates that ^18^F-FMCH is a potential tracer for monitoring the atherosclerotic vascular inflammation in diabetes.

## Additional file

10.1186/s12933-016-0340-6 Supplemental data.

## Abbreviations

% IA/g: percentage of injected radioactivity dose per gram of tissue
^18^F-FDG: 2-deoxy-2-[^18^F]-fluoro-*d*-glucose
^18^F-FMCH: ^18^F-fluoromethylcholine
CT: computed tomography
CVD: cardiovascular disease
H&E: hematoxylin–eosin staining
IFN-γ: interferon-γ
IGF-II: insulin-like growth factor II
IL-6: interleukin-6
iNOS: inducible nitric oxide synthase
LDL: low-density lipoprotein
MCP-1: monocyte chemoattractant protein-1
MRC-1: mannose receptor C-type 1
PET: positron emission tomography
PLTP: phospholipid transfer protein
PON-1: paraoxonase-1
PSL/mm^2^: photo-stimulated luminescence per square millimeter
RANTES: cytokine regulated on activation, normal T cell expressed and secreted
ROI: region of interest
T2DM: type 2 diabetes mellitus

## References

[1] Seshasai SRK, Kaptoge S, Thompson A, Di Angelantonio E, Gao P, Sarwar N (2011). Diabetes mellitus, fasting glucose, and risk of cause-specific death. N Engl J Med.

[2] Pasterkamp G (2013). Methods of accelerated atherosclerosis in diabetic patients. Heart.

[3] Libby P, Theroux P (2005). Pathophysiology of coronary artery disease. Circulation.

[4] Rudd JHF, Narula J, Strauss HW, Virmani R, Machac J, Klimas M (2010). Imaging atherosclerotic plaque inflammation by fluorodeoxyglucose with positron emission tomography. Ready for prime time?. J Am Coll Cardiol.

[5] Moore KJ, Tabas I (2011). Macrophages in the pathogenesis of atherosclerosis. Cell.

[6] Kim TN, Kim S, Yang SJ, Yoo HJ, Seo JA, Kim SG (2010). Vascular inflammation in patients with impaired glucose tolerance and type 2 diabetes: analysis with ^18^F-fluorodeoxyglucose positron emission tomography. Circ Cardiovasc Imaging..

[7] Bucerius J, Mani V, Moncrieff C, Rudd JHF, MacHac J, Fuster V (2012). Impact of noninsulin-dependent type 2 diabetes on carotid wall ^18^F-fluorodeoxyglucose positron emission tomography uptake. J Am Coll Cardiol.

[8] Tahara N, Kai H, Yamagishi S, Mizoguchi M, Nakaura H, Ishibashi M (2007). Vascular inflammation evaluated by [^18^F]-fluorodeoxyglucose positron emission tomography is associated with the metabolic syndrome. J Am Coll Cardiol.

[9] Kang S, Kyung C, Park JS, Kim S, Lee S-P, Kim MK (2014). Subclinical vascular inflammation in subjects with normal weight obesity and its association with body fat: an ^18^F-FDG-PET/CT study. Cardiovasc Diabetol..

[10] Rudd JHF, Warburton EA, Fryer TD, Jones HA, Clark JC, Antoun N (2002). Imaging atherosclerotic plaque inflammation with [^18^F]-fluorodeoxyglucose positron emission tomography. Circulation.

[11] Ogawa M, Nakamura S, Saito Y, Kosugi M, Magata Y (2012). What can be seen by ^18^F-FDG PET in atherosclerosis imaging? The effect of foam cell formation on ^18^F-FDG uptake to macrophages in vitro. J Nucl Med.

[12] Tawakol A, Singh P, Mojena M, Pimentel-Santillana M, Emami H, MacNabb M (2015). HIF-1 and PFKFB3 mediate a tight relationship between proinflammatory activation and anerobic metabolism in atherosclerotic macrophages. Arterioscler Thromb Vasc Biol.

[13] Folco EJ, Sheikine Y, Rocha VZ, Christen T, Shvartz E, Sukhova GK (2011). Hypoxia but not inflammation augments glucose uptake in human macrophages: implications for imaging atherosclerosis with ^18^fluorine-labeled 2-deoxy-d-glucose positron emission tomography. J Am Coll Cardiol.

[14] Pedersen SF, Græbe M, Hag AMF, Højgaard L, Sillesen H (2013). ^18^F-FDG imaging of human atherosclerotic carotid plaques reflects gene expression of the key hypoxia marker HIF-1α. Am J Nucl Med Mol Imaging..

[15] Tekabe Y, Luma J, Einstein AJ, Sedlar M, Li Q, Schmidt AM (2010). A novel monoclonal antibody for RAGE-directed imaging identifies accelerated atherosclerosis in diabetes. J Nucl Med.

[16] Rominger A, Saam T, Vogl E, Ubleis C, la Fougère C, Förster S (2010). In vivo imaging of macrophage activity in the coronary arteries using ^68^Ga-DOTATATE PET/CT: correlation with coronary calcium burden and risk factors. J Nucl Med.

[17] Gaemperli O, Shalhoub J, Owen DRJ, Lamare F, Johansson S, Fouladi N (2012). Imaging intraplaque inflammation in carotid atherosclerosis with ^11^C-PK11195 positron emission tomography/computed tomography. Eur Heart J.

[18] Treglia G, Giovannini E, Di Franco D, Calcagni ML, Rufini V, Picchio M (2012). The role of positron emission tomography using carbon-11 and fluorine-18 choline in tumors other than prostate cancer: a systematic review. Ann Nucl Med.

[19] Krause BJ, Souvatzoglou M, Treiber U (2013). Imaging of prostate cancer with PET/CT and radioactively labeled choline derivates. Urol Oncol Semin Orig Investig..

[20] Roivainen A, Parkkola R, Yli-Kerttula T, Lehikoinen P, Viljanen T, Möttönen T (2003). Use of positron emission tomography with methyl-^11^C-choline and 2-^18^F-fluoro-2-deoxy-d-glucose in comparison with magnetic resonance imaging for the assessment of inflammatory proliferation of synovium. Arthritis Rheum.

[21] Wyss MT, Weber B, Honer M, Späth N, Ametamey SM, Westera G (2004). ^18^F-choline in experimental soft tissue infection assessed with autoradiography and high-resolution PET. Eur J Nucl Med Mol Imaging.

[22] Laitinen IEK, Luoto P, Någren K, Marjamäki PM, Silvola JMU, Hellberg S (2010). Uptake of ^11^C-choline in mouse atherosclerotic plaques. J Nucl Med.

[23] Matter CM, Wyss MT, Meier P, Späth N, Von Lukowicz T, Lohmann C (2006). ^18^F-choline images murine atherosclerotic plaques ex vivo. Arterioscler Thromb Vasc Biol.

[24] Sarda-Mantel L, Alsac JM, Boisgard R, Hervatin F, Montravers F, Tavitian B (2012). Comparison of ^18^F-fluoro-deoxy-glucose, ^18^F-fluoro-methyl-choline, and ^18^F-DPA714 for positron-emission tomography imaging of leukocyte accumulation in the aortic wall of experimental abdominal aneurysms. J Vasc Surg.

[25] Kato K, Schober O, Ikeda M, Schäfers M, Ishigaki T, Kies P (2009). Evaluation and comparison of ^11^C-choline uptake and calcification in aortic and common carotid arterial walls with combined PET/CT. Eur J Nucl Med Mol Imaging.

[26] Bucerius J, Schmaljohann J, Böhm I, Palmedo H, Guhlke S, Tiemann K (2008). Feasibility of ^18^F-fluoromethylcholine PET/CT for imaging of vessel wall alterations in humans-First results. Eur J Nucl Med Mol Imaging.

[27] Heinonen SE, Leppänen P, Kholová I, Lumivuori H, Häkkinen SK, Bosch F (2007). Increased atherosclerotic lesion calcification in a novel mouse model combining insulin resistance, hyperglycemia, and hypercholesterolemia. Circ Res.

[28] Véniant MM, Zlot CH, Walzem RL, Pierotti V, Driscoll R, Dichek D (1998). Lipoprotein clearance mechanisms in LDL receptor-deficient “apo-B48- only” and “apo-B100-only” mice. J Clin Invest..

[29] Kolthammer JA, Corn DJ, Tenley N, Wu C, Tian H, Wang Y (2011). PET imaging of hepatocellular carcinoma with ^18^F-fluoroethylcholine and ^11^C-choline. Eur J Nucl Med Mol Imaging.

[30] Silvola JMU, Saraste A, Laitinen I, Savisto N, Laine VJO, Heinonen SE (2011). Effects of age, diet, and type 2 diabetes on the development and FDG uptake of atherosclerotic plaques. JACC Cardiovasc Imaging..

[31] Haukkala J, Laitinen I, Luoto P, Iveson P, Wilson I, Karlsen H (2009). ^68^Ga-DOTA-RGD peptide: biodistribution and binding into atherosclerotic plaques in mice. Eur J Nucl Med Mol Imaging.

[32] Rinne P, Silvola JMU, Hellberg S, Ståhle M, Liljenbäck H, Salomäki H (2014). Pharmacological activation of the melanocortin system limits plaque inflammation and ameliorates vascular dysfunction in atherosclerotic mice. Arterioscler Thromb Vasc Biol.

[33] Hong HC, Hwang SY, Park S, Ryu JY, Choi HY, Yoo HJ (2015). Implications of pericardial, visceral and subcutaneous adipose tissue on vascular Inflammation measured using ^18^FDG-PET/CT. PLoS One.

[34] Heinonen SE, Merentie M, Hedman M, Mäkinen PI, Loponen E, Kholová I (2011). Left ventricular dysfunction with reduced functional cardiac reserve in diabetic and non-diabetic LDL-receptor deficient apolipoprotein B100-only mice. Cardiovasc Diabetol..

[35] De Vries R, Dallinga-Thie GM, Smit AJ, Wolffenbuttel BHR, Van Tol A, Dullaart RPF (2006). Elevated plasma phospholipid transfer protein activity is a determinant of carotid intima-media thickness in type 2 diabetes mellitus. Diabetologia.

[36] Zhang K, Liu X, Yu Y, Luo T, Wang L, Ge C (2014). Phospholipid transfer protein destabilizes mouse atherosclerotic plaque. Arterioscler Thromb Vasc Biol.

[37] Stamler J, Wentworth D, Neaton JD (1986). Is relationship between serum cholesterol and risk of premature death from coronary heart disease continuous and graded? Findings in 356,222 primary screenees of the multiple risk factor intervention trial (MRFIT). JAMA.

[38] Schuett H, Oestreich R, Waetzig GH, Annema W, Luchtefeld M, Hillmer A (2012). Transsignaling of interleukin-6 crucially contributes to atherosclerosis in mice. Arterioscler Thromb Vasc Biol.

[39] Koh KK, Park SM, Quon MJ (2008). Leptin and cardiovascular disease: response to therapeutic interventions. Circulation.

[40] Ducimetiere P, Eschwege E, Papoz L, Richard JL, Claude JR, Rosselin G (1980). Relationship of plasma insulin levels to the incidence of myocardial infarction and coronary heart disease mortality in a middle-aged population. Diabetologia.

[41] Ridker PM, Rifai N, Stampfer MJ, Hennekens CH (2000). Plasma concentration of interleukin-6 and the risk of future myocardial infarction among apparently healthy men. Circulation.

[42] Veillard NR, Kwak B, Pelli G, Mulhaupt F, James RW, Proudfoot A (2004). Antagonism of RANTES receptors reduces atherosclerotic plaque formation in mice. Circ Res.

[43] Kim DS, Burt AA, Ranchalis JE, Vuletic S, Vaisar T, Li W-F (2015). Plasma phospholipid transfer protein (PLTP) activity inversely correlates with carotid artery disease: effects of paraoxonase 1 enzyme activity and genetic variants on PLTP activity. J Lipid Res.

[44] Durrington PN, Mackness B, Mackness MI (2001). Paraoxonase and atherosclerosis. Arterioscler Thromb Vasc Biol.

[45] Tward A, Xia Y-R, Wang X-P, Shi Y-S, Park C, Castellani LW (2002). Decreased atherosclerotic lesion formation in human serum paraoxonase transgenic mice. Circulation.

[46] Buettner C, Rudd JHF, Fayad ZA (2011). Determinants of FDG uptake in atherosclerosis. JACC Cardiovasc Imaging..

