# Supplementary material for: Type 2 diabetes enhances arterial uptake of choline in atherosclerotic mice: an imaging study with positron emission tomography tracer 18F-fluoromethylcholine
Source: Cardiovasc Diabetol. 2016 Feb 6;15:26. doi: 10.1186/s12933-016-0340-6 (PMC4744438; doi:10.1186/s12933-016-0340-6)
Supplement: Supplementary file 1 — 10.1186/s12933-016-0340-6 Supplemental data. [file 12933_2016_340_MOESM1_ESM.pdf]

Type 2 diabetes enhances arterial uptake of choline in atherosclerotic mice: An imaging study with positron emission tomography tracer ^{18}F-fluoromethylcholine

Sanna Hellberg[1], Johanna M.U. Silvola[1], Max Kiugel[1], Heidi Liljenbäck[1,2], Olli Metsälä[1], Tapio Viljanen[1], Jari Metso[3], Matti Jauhiainen[3], Pekka Saukko[4], Pirjo Nuutila[1], Seppo Ylä-Herttuala[5,6], Juhani Knuuti[1], Anne Roivainen[1,2], Antti Saraste[1,7]

1 University of Turku, Turku PET Centre, Turku, Finland

2 University of Turku, Turku Center for Disease Modeling, Turku, Finland

3 National Institute for Health and Welfare, Genomics and Biomarkers Unit, Helsinki, Finland

4 University of Turku, Department of Pathology and Forensic Medicine, Turku, Finland

5 University of Eastern Finland, A.I. Virtanen Institute for Molecular Sciences, Kuopio, Finland

6 Kuopio University Hospital, Science Service Center, Kuopio, Finland

7 Turku University Hospital, Heart Center, Turku, Finland

ADDITIONAL FILE 1: SUPPLEMENTAL DATA

SUPPLEMENTAL MATERIALS AND METHODS

In vivo stability of ^{18}F-FMCH

To determine *in vivo* radiometabolism, blood samples were taken from 21 mice (eight LDLR$^{-/-}$ApoB$^{100/100}$, eight IGF-II/LDLR$^{-/-}$ApoB$^{100/100}$ and five C57Bl/6N healthy controls) at sacrifice. Plasma was separated with centrifugation (2100 G, 5 min) and proteins were precipitated with acetonitrile. High performance liquid chromatography coupled with on-line radioactivity detector (Radio-HPLC) was performed with Phenomenex Partisil 10 µm SCX, 250 x 4.6 mm column, as described in [1].

Additional animal experiments: uptake of ^{18}F-FMCH in more advanced atherosclerosis and in fasting state

An additional group of older atherosclerotic mice with diabetes (IGF-II/LDLR$^{-/-}$ApoB$^{100/100}$) that were fed with a high-fat diet of a longer duration (age 8-10 months, high-fat diet 6 months, *n*=9, Supplemental Table 1) were studied to evaluate the ^{18}F-FMCH uptake in more advanced stages of atherosclerosis. The mice were studied for ^{18}F-FMCH biodistribution, autoradiography and histology in a similar manner as the other mice in the study. The results were compared with 6-month-old IGF-II/LDLR$^{-/-}$ApoB$^{100/100}$ mice and with C57BL/6N controls (where applicable).

The effects of fasting were studied in a small cohort of LDLR$^{-/-}$ApoB$^{100/100}$ mice (age 6-7 months, high-fat diet 4 months, *n*=3, Supplemental Table 1). The mice were fasted for 4 hours and studied for *ex vivo* biodistribution and biomarkers in the plasma as described in the main article.

Immunohistochemical stainings

Aortic root sections were immunostained for macrophages (Mac-3), M1 and M2 polarization markers (iNOS and MRC-1), and CD36. For the Mac-3 staining, the paraffin sections were first de-paraffinized and rehydrated. After washes and bovine serum albumin (BSA) blocking, the sections were incubated with primary antibody (Anti-Mac-3 M3/84, 1:500, BD Pharmingen) for one hour followed by peroxidase treatment and washes. Rat-on-Mouse HRP-polymer RT 517 kit (Biocare Medical, Concord, CA, USA) was utilized according to the manufacturer's instructions, followed by chromogen (DAB, Dako K3468) and counterstaining with Mayer's hematoxylin. Consecutive sections were stained for iNOS and MRC-1. The paraffin sections were de-paraffinized, rehydrated and pre-incubated in 10 mM citric acid. After washes and BSA blocking, the primary antibody (anti-iNOS ab15323, 1:200, or anti-MRC-1 ab64693, 1:500, Abcam, Cambridge, UK) was added and incubated for one hour. Secondary antibody (Dako EnVision anti-rabbit K4003) was added and incubated for 30 min after washing and H_2O_2 treatment. The sections were again washed, followed by the addition of chromogen and counterstaining as described above. For CD36, the protocol was similar, except for the pre-incubation, which was performed by boiling in 10 mM tris-EDTA buffer. The primary antibody was anti-CD36 ab 80978, 1:500, (Abcam). The Ki-67 stainings were performed

on longitudinally cut aortic cryosections. The sections were first fixed in 10 % formalin and pre-incubated in 10 mM boiling citric acid. After washes and blocking with 5 % goat serum in 3 % BSA, the primary antibody (monoclonal rat anti-mouse clone TEC-3, M7249, 1:1000, Dako) was incubated overnight. Secondary antibody (polyclonal rabbit anti-rat E0468, 1:200, Dako) was incubated for 30 minutes after washes and H_2O_2 treatment, followed by tertiary antibody (EnVision anti-rabbit, K4003, Dako). The detection and counterstaining was performed similarly as described above.

Plasma biomarker measurements

Phospholipids were analyzed using the Phospholipids B kit (Wako Chemicals, Neuss, Germany) or Pureauto S PL kit (Daiichi Pure Chemicals, Tokyo, Japan), triglycerides using the Triglycerides GPO-PAP kit (Roche Diagnostics, Basel, Switzerland) and total cholesterol using the Cholesterol CHOD-PAP kit (Roche Diagnostics, Basel, Switzerland). In addition, phospholipid transfer protein (PLTP) and paraoxonase-1 (PON-1) activities were measured. PLTP activity (mmol $ml^{-1}h^{-1}$) was determined with a radiometric method, as described in [2]. PON-1 activity (µmol/min) was measured with a chromogenic method [3]. Briefly, in the PON-1 assay, paraoxon (diethyl-p-nitrophenylphosphate, Sigma-Aldrich, St. Louis, MO, USA) was used as a substrate. PON converts paraoxon to p-nitrophenol, a yellow compound that can be measured spectrophotometrically at 405 nm. The intra-assay and inter-assay coefficients of variation for PON measurements are 10% and 7%, respectively. The levels of IL-6 IL-1β, RANTES, MCP-1 and IFN-γ were measured using a Luminex assay according to the manufacturer's instructions (MILLIPLEX MAP Mouse Cytokine/Chemokine Magnetic Bead Panel, Merck Millipore, Billerica, MA, USA). Another Luminex assay was utilized for the measurement of C-peptide, glucagon, insulin and leptin (MILLIPLEX MAP Mouse Metabolic Hormone Magnetic Bead Panel, Merck Millipore, Billerica, MA, USA). Glucose was measured either from fresh whole blood with a glucometer (One Touch UltraEasy, LifeScan, Inc., Milpitas, CA, USA), calibrated to measure plasma levels of glucose, or from frozen and thawn plasma with a glucose analyzer (GM9, Analox Instruments, London, UK).

Plasma pools for the lipoprotein fractioning were derived from 11 LDLR$^{-/-}$ApoB$^{100/100}$ mice, 10 IGF-II/LDLR$^{-/-}$ApoB$^{100/100}$ mice and 7 C57BL/6N mice. The lipid profiles were measured with fast-performance liquid chromatography method, as described earlier [4]. The lipoproteins in the mouse plasma were fractionated and classified as very low-density lipoproteins (VLDL), intermediate- and low-density lipoproteins (IDL-LDL), and high-density lipoproteins (HDL). The concentration of cholesterol, triglycerides and phospholipids was measured in each fraction and expressed as mmol/l.

SUPPLEMENTAL RESULTS

In vivo stability of ^{18}F-FMCH

Intravenously administered ^{18}F-FMCH showed rapid *in vivo* radiotracer metabolism with no differences between the mouse strains. Twenty minutes after injection, 7.9 ± 0.78 % of plasma total radioactivity originated from the intact tracer (radio-HPLC retention time 4.0 min). In the urine, 40 ± 2.4 % of the radioactivity was excreted as intact tracer. The main radioactive metabolite in mice plasma was ^{18}F-betaine (retention time 2.9 min). Representative radio-HPLC chromatograms are presented in Supplemental Figure 1.

Aged diabetic mice represent larger and less inflamed plaques with lower ^{18}F-FMCH uptake

The group of aged (8-10-month-old) IGF-II/LDLR$^{-/-}$ApoB$^{100/100}$ mice showed larger atherosclerotic plaques as compared with the 6-month-old mice of the same genotype (Supplemental Figure 2 and Supplemental Table 1). All of the older IGF-II/LDLR$^{-/-}$ApoB$^{100/100}$ mice showed calcified areas in plaques. The intima-to-media ratio was significantly higher (4.3 ± 0.49, $p<0.001$), whereas the percentages of Mac-3, iNOS and MRC-1 staining in plaques were lower than in the 6-month-old IGF-II/LDLR$^{-/-}$ApoB$^{100/100}$ mice (11 ± 0.55, $p=0.011$; 8.0 ± 0.23, $p=0.006$; 23 ± 2.4, $p=0.026$, respectively). The ^{18}F-FMCH whole-body distribution in the aged mice (Supplemental Table 2) was very similar to the 6-month-old mice of the same genotype (Table 3 in the main article). The only significant difference was observed in the lung uptake, which was lower in the

aged mice (9.4 ± 1.1 vs. 13 ± 1.0 %IA/g, p= 0.031). The aortic uptake of aged IGF-II/LDLR$^{-/-}$ApoB$^{100/100}$ mice was 1.7 ± 0.21 %IA/g, and aorta-to-blood ratio 3.8 ± 0.79 (difference to controls or 6-month-old mice of the same strain was not significant). In the autoradiography analysis, the plaque uptake was 170 ± 15 PSL/mm^2 and plaque-to-wall ratio was 2.2 ± 0.088, which both were significantly lower than the corresponding values in the 6-month-old age group (p=0.032 and p=0.0014, respectively).

Fasting has no significant effect on ^{18}F-FMCH uptake or plasma markers

The mice that fasted for 4 hours before ^{18}F-FMCH injection showed very similar characteristics as the non-fasted mice of the same strain (Supplemental Table 1 and Table 1 of the main article). There were no differences in ^{18}F-FMCH uptake between different tissues (Supplemental Table 2 and Table 3 of the main article) or in the measured markers in the plasma, except for a subtle change in PLTP activity (Supplemental Table 3).

Lipoprotein fraction measurements

The lipoprotein distributions were determined from pooled plasma samples in IGF-II/LDLR$^{-/-}$ApoB$^{100/100}$, LDLR$^{-/-}$ApoB$^{100/100}$ and C57BL/6N mice. The levels of cholesterol, triglycerides and phospholipids in very low-density lipoproteins (VLDL) and intermediate- and low-density lipoproteins (IDL-LDL) were higher in both of the atherosclerotic mouse models than in controls (Supplemental Figure 3). The lipoprotein profile was shifted towards higher levels of VLDL-associated cholesterol and phospholipids, and lower levels of these same lipids associated with IDL-LDL in IGF-II/LDLR$^{-/-}$ApoB$^{100/100}$ mice as compared with LDLR$^{-/-}$ApoB$^{100/100}$ mice. The high-density lipoprotein (HDL) associated cholesterol levels had no differences between strains, whereas the HDL-associated phospholipid levels tended to be lower in the IGF-II/LDLR$^{-/-}$ApoB$^{100/100}$ mice. The differences, however, were modest and measured from only one pool of plasma from each strain, so no direct conclusions can be made.

SUPPLEMENTAL TABLES

SUPPLEMENTAL TABLE 1. Characteristics of the additional mice studied with ^{18}F-FMCH.

	Aged IGFII/LDLR$^{-/-}$ ApoB$^{100/100}$ mice	Fasted LDLR$^{-/-}$ ApoB$^{100/100}$ mice
Number (m/f)	9 (4/5)	3 (3/0)
Weight (g)	35 ± 6.8	38 ± 1.2
Age (months)	8.7 ± 1.4	6.5 ± 0.058
High-fat diet (months)	6.2 ± 0.29	4.4 ± 0.0
Injected radioactivity (MBq)	9.6 ± 1.4	9.1 ± 0.48
Autoradiography (n)	9	0
Plasma glucose (mmol/l)	19 ± 2.5[a]	15 ± 0.42[a]
Intima-to-media ratio (plaque size)	4.3 ± 1.1[b]	3.5 ± 2.0

The data are expressed as mean ± SD.

[a] *n*=2

[b] *n*=5

SUPPLEMENTAL TABLE 2. The distribution of ^{18}F-FMCH in tissues in fasted LDLR$^{-/-}$ApoB$^{100/100}$ mice and aged IGF-II/LDLR$^{-/-}$ApoB$^{100/100}$ mice. Results are expressed as percentage of injected radioactivity per gram of tissue (%IA/g), mean ± SEM.

Tissue	Aged IGF-II/LDLR$^{-/-}$ApoB$^{100/100}$ mice (n=9)			Fasted LDLR$^{-/-}$ApoB$^{100/100}$ mice (n=3)		
Aorta	1.7	±	0.21	1.6	±	0.2
Blood	0.55	±	0.093	0.76	±	0.13
Intestine	7.5	±	1.3	7.5	±	0.6
Kidney	38	±	6.6	54	±	4.8
Liver	15	±	1.6	13	±	3
Lungs	9.4	±	1.1	9.9	±	0.3
Muscle	0.93	±	0.092	0.82	±	0.09
Myocardium	6.7	±	0.81	6.3	±	0.2
Pancreas	7.1	±	0.62	9.3	±	0.3
Spleen	5.3	±	0.73	6.7	±	0.5
Urine	23	±	9.1		NA	
White adipose tissue	0.33	±	0.040	0.36	±	0.02

No statistically significant differences were observed between fasted and non-fasted mice (results in the main article). ^{18}F-FMCH uptake in lungs was lower in aged IGF-II/LDLR$^{-/-}$ApoB$^{100/100}$ mice as compared to 6-month old mice of the same strain (results in the main article). NA=not analyzed.

SUPPLEMENTAL TABLE 3. Plasma levels of biomarkers in atherosclerotic and control mice. Results are expressed as mean ± SEM.

	$LDLR^{-/-}ApoB^{100/100}$	$IGF\text{-}II/LDLR^{-/-}$ $ApoB^{100/100}$	C57BL/6N	Fasted $LDLR^{-/-}$ $ApoB^{100/100}$
Total cholesterol (mmol/l)	31 ± 1.3	36 ± 3.7	1.8 ± 0.11[a]	31 ± 3.9
Phospholipids (mmol/l)	7.5 ± 0.50	7.4 ± 0.41	1.7 ± 0.14[a]	7.3 ± 0.76
Triglycerides (mmol/l)	2.2 ± 0.23	2.1 ± 0.28	0.6 ± 0.12[a]	2.1 ± 0.21
PLTP (mmol ml^{-1} h^{-1})	42 ± 1.3	45 ± 2.2	22 ± 1.4* [a]	49 ± 2.8[b]
PON-1 (µmol/min)	77 ± 5.0	74 ± 5.1	69 ± 7.4	81 ± 9.7
C-peptide (pg/ml)	1200 ± 250	3000 ± 670	1800 ± 320	1800 ± 220
Glucagon (pg/ml)	160 ± 67	70 ± 15	180 ± 120	50 ± 18
Insulin (pg/ml)	950 ± 150	2900 ± 800	2700 ± 860	1500 ± 510
Leptin (pg/ml)	5600 ± 760	6200 ± 1300	4100 ± 1100	8000 ± 1200
IFN-γ (pg/ml)	18 ± 5.8	3.4 ± 1.1	11 ± 5.6	10 ± 7.2
IL-1β (pg/ml)	45 ± 26	15 ± 7.7	1.9 ± 0.35	61 ± 35
IL-6 (pg/ml)	26 ± 8.4	88 ± 28	13 ± 7.6	24 ± 0.93
RANTES (pg/ml)	18 ± 4.2	20 ± 8.6	8.4 ± 3.1	32 ± 2.1

[a]A significant difference as compared with the $LDLR^{-/-}ApoB^{100/100}$ and $IGF\text{-}II/LDLR^{-/-}ApoB^{100/100}$ mice.

[b]A significant difference as compared with $LDLR^{-/-}ApoB^{100/100}$ mice.

SUPPLEMENTAL FIGURES

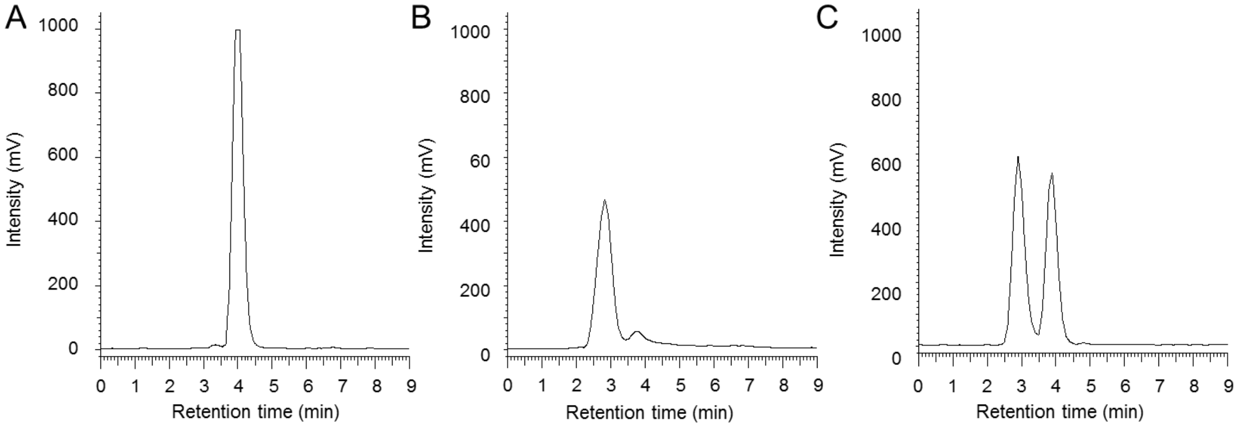

SUPPLEMENTAL FIGURE 1. *In vivo* radiometabolism of ^{18}F-FMCH in mice as determined by radio-HPLC. A: Chromatogram of ^{18}F-FMCH. B: Representative chromatogram of a LDLR$^{-/-}$ApoB$^{100/100}$ mouse plasma sample obtained 20 minutes post-injection, showing one major metabolite and small portion of non-metabolized ^{18}F-FMCH. On average, 7.9 ± 0.78 % of ^{18}F-FMCH was intact in the plasma. C: Representative HPLC-gram of urine sample obtained 20 minutes post-injection. In the urine, 40 ± 2.4 % was excreted as intact tracer. No differences were observed in the ^{18}F-FMCH radiometabolism between the mouse strains.

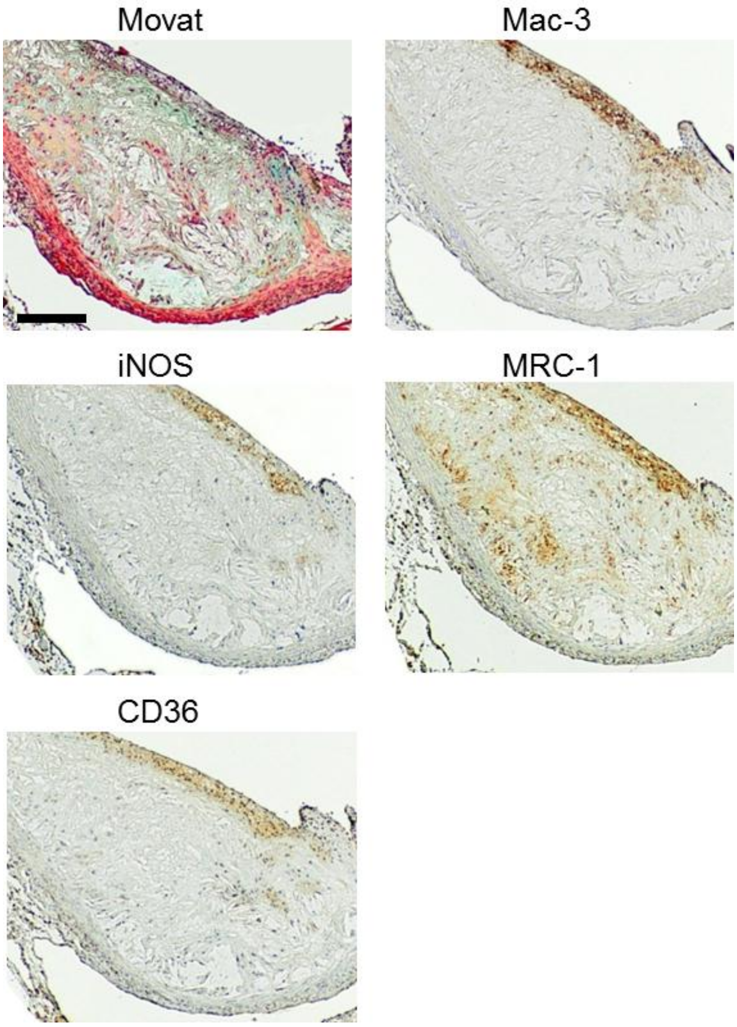

SUPPLEMENTAL FIGURE 2. Histology and immunohistochemistry of atherosclerotic plaque in aged diabetic IGF-II/LDLR$^{-/-}$ApoB$^{100/100}$ mice. The aortic roots represent larger atherosclerotic plaques with less macrophages as compared with the 6-month-old mice shown in Figure 1 of the main article. Mac-3: activated macrophages; iNOS: M1-polarized macrophages, MRC-1: M2-polarized macrophages; CD36: scavenger receptor. Scale bar 100 µm.

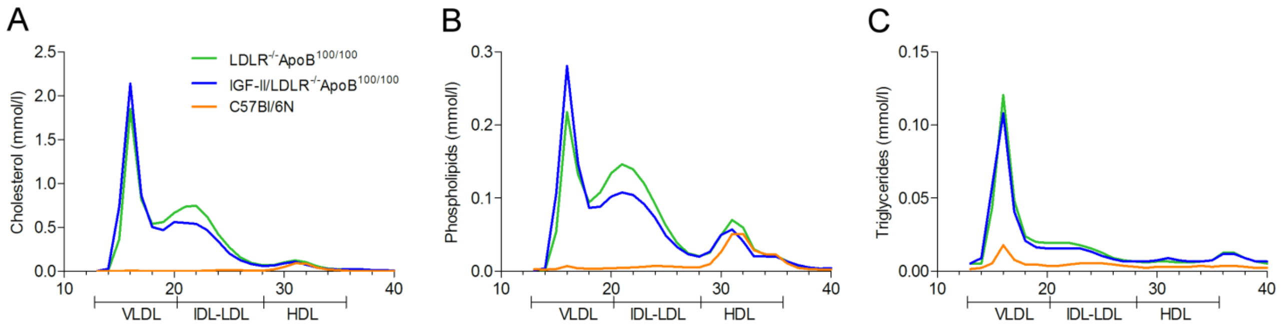

SUPPLEMENTAL FIGURE 3. Lipoprotein lipid distribution in the plasma of non-diabetic LDLR$^{-/-}$ApoB$^{100/100}$ and diabetic IGF-II/LDLR$^{-/-}$ApoB$^{100/100}$ hypercholesterolemic mice and C57Bl/6N healthy control mice. A: Cholesterol, B: Phospholipids, C: Triglycerides. The lipoprotein profile of C57Bl/6N mice is different from the hypercholesterolemic models. In IGF-II/LDLR$^{-/-}$ApoB$^{100/100}$ mice, the lipoprotein profile is shifted towards higher levels of VLDL-associated cholesterol and phospholipids, and lower levels of these same lipids associated with IDL-LDL as compared with LDLR$^{-/-}$ApoB$^{100/100}$ mice.

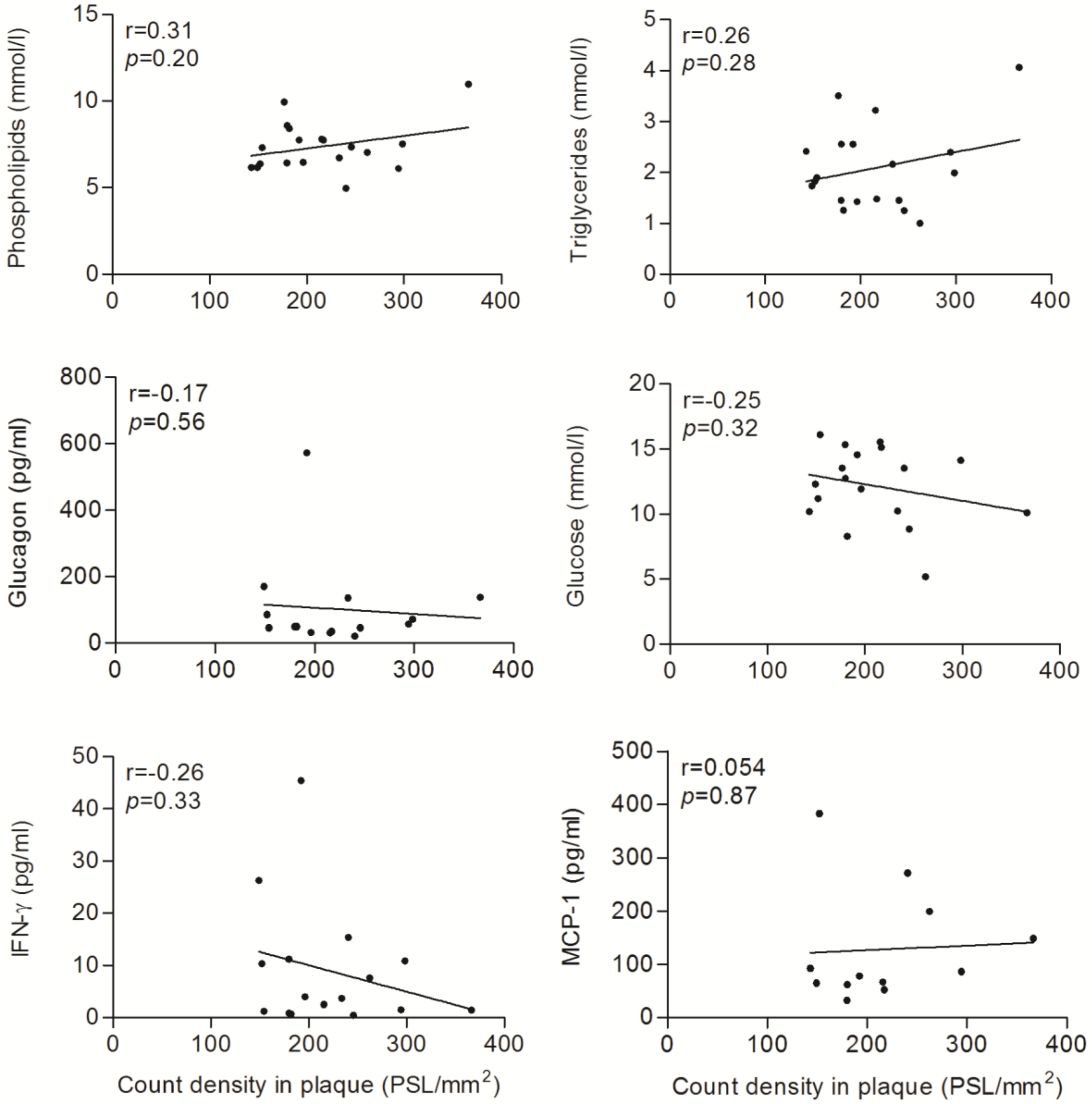

SUPPLEMENTAL FIGURE 4.Non-significant correlations between the ^{18}F-FMCH uptake and the plasma levels of lipids, metabolic markers and cytokines. The data are pooled from 6-month-old LDLR$^{-/-}$ApoB$^{100/100}$ and IGF-II/LDLR$^{-/-}$ApoB$^{100/100}$ mice.

SUPPLEMENTAL REFERENCES

1. Roivainen A, Parkkola R, Yli-Kerttula T, et al. (2003) Use of positron emission tomography with methyl-^{11}C-choline and 2-^{18}F-fluoro-2-deoxy-D-glucose in comparison with magnetic resonance imaging for the assessment of inflammatory proliferation of synovium. Arthritis Rheum 48:3077–3084
2. Jauhiainen M, Ehnholm C (2005) Determination of human plasma phospholipid transfer protein mass and activity. Methods 36:97–101
3. Kleemola P, Freese R, Jauhiainen M, et al. (2002) Dietary determinants of serum paraoxonase activity in healthy humans. Atherosclerosis 160:425–432
4. Yan D, Jauhiainen M, Hildebrand RB, et al. (2007) Expression of human OSBP-related protein 1L in macrophages enhances atherosclerotic lesion development in LDL receptor-deficient mice. Arterioscler Thromb Vasc Biol 27:1618–1624
